# Penetration of Cement Pastes into Particle-Beds: A Comparison of Penetration Models

**DOI:** 10.3390/ma14020389

**Published:** 2021-01-14

**Authors:** Daniel Weger, Alexandre Pierre, Arnaud Perrot, Thomas Kränkel, Dirk Lowke, Christoph Gehlen

**Affiliations:** 1 Chair of Materials Science and Testing, Centre for Building Materials (CBM), Technical University of Munich, 81245 Munich, Germany; gehlen@tum.de; 2 L2MGC, EA4114, CY Cergy Paris Université, 95031 Cergy-Pontoise, France; alexandre.pierre@cyu.fr; 3 Institut de Recherche Dupuy de Lôme (IRDL), Université de Bretagne Sud, UMR CNRS 6027, IRDL, 56100 Lorient, France; arnaud.perrot@univ-ubs.fr; 4 Institute of Building Materials, Concrete Construction and Fire Safety (iBMB), Technische Universität Braunschweig, 38106 Braunschweig, Germany; d.lowke@ibmb.tu-bs.de

**Keywords:** 3D printing, particle-bed, selective paste intrusion, concrete, cement, penetration, analytical model

## Abstract

For the selective paste intrusion (SPI) method, thin layers of aggregate are locally bound by cement paste where the structure shall arise. After completion of the printing process, the structure is excavated from the particle-bed and the unbound particles are removed. However, for a sufficient layer bonding and shape accuracy, the rheology of the cement paste must be adapted to the flow resistance of the particle-bed. For practical application, that means mostly time and material consuming “trial and error” tests. To prevent that, analytical models can help to predict the penetration of the cement paste. This paper presents four analytical models to calculate the penetration depth of a cement paste into a particle packing. Based on Darcy’s law, an already existing model is slightly modified (model A+) and a generalized (model C), an advanced generalized (model D) as well as a simplified model (model B/B+) are developed. Compared to conducted tests on the penetration depth, model B showed good accuracy (deviation <1.5 mm) for pastes with a yield stress ≥8.2 Pa, model A+/B+/C for ≥ 5.4 Pa and model D even for <5.4 Pa. Finally, an application guide for each model for practical use will be given.

## 1. Introduction

Due to the growing scarcity of resources, especially of aggregates for concrete production, and the high energy requirements as well as CO_2_ emissions of cement production, lightweight construction and material saving are becoming increasingly important [1,2,3].

Consequently, the design principles in civil engineering must move away from a simple, compact construction to a more complex design that needs less material displaying multifunctionalities. First projects point out the enormous potential of saving resources by using additive manufacturing processes [4,5,6,7,8]. In addition, there are further ecological and economic advantages, such as faster production, elimination of formwork (and thus savings in waste and costs) [9,10,11,12,13,14,15] and the possibility of realizing new ways in the design of buildings (e.g., the principle that form follows force) [16,17,18,19] or new reinforcement concepts [20,21,22,23].

Many of the digital construction projects using additive manufacturing deal with depositing processes, also called extrusion. These enable one to produce large concrete components within a short time. In addition, there is usually no limitation by a construction space. A drawback of the depositing process is the lack of freedom of form, since strong overhangs can only be realized with the help of supporting structures [24,25,26]. An exception is shotcrete 3D printing technology, which enables also strong overhanging structures [27]. However, these techniques could require the use of a combination of additives and admixtures which increase the cost and the complexity of the mix-design of the material.

**Figure 1:**
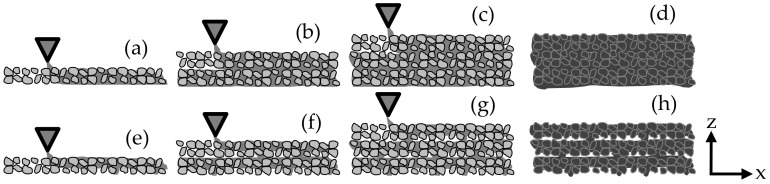
Production process of Selective (Cement) Paste Intrusion (SPI) with complete layer bonding/filling of voids (**a**–**d**) and with incomplete layer bonding/filling of voids (**e**–**h**) [26].

Overhangs of almost any complexity combined with much higher surface resolutions can be realized with selective binding processes [28], which were first used in 1995 by Pegna [29,30] for the production of concrete elements. A variant (which is applied in this paper) is the Selective (Cement) Paste Intrusion—SPI method [31,32]. Here, a particle-bed of aggregate is bound with cement paste where the component is to be produced; see Figure 1a–d.

**Figure 2:**
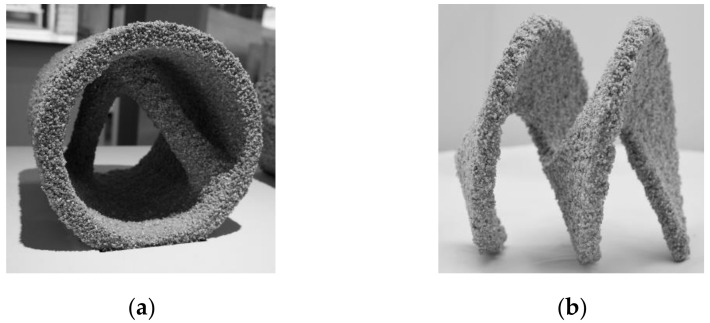
(**a**) SPI manufactured tube with internal double bracing; (**b**) SPI manufactured Helix (wing thickness 0.015 m); picture (**b**) C. Matthaeus.

Due to the supporting function of the unbound particles, almost any overhanging construction with high surface resolution, high strength (up to 78 MPa) and high durability as well as density comparable to ordinary concrete (by filling up a present particle network) [26,33,34,35] can be created, Figure 2.

In contrast, the selective binding method Selective Cement Activation (SCA) uses an aggregate–binder (cement) mixture which is locally hardened by a water-based activator [28,36,37,38,39]. The SCA method has already been in focus for building lunar outposts [40] and can be used for the production of structures made of lightweight concrete [41].

SCA offers a high surface resolution [42,43]. The surface resolution of SPI is in between the extrusion depositing processes and SCA. However, compared to SCA, SPI can achieve a faster construction speed due its coarser particles and its associated higher layer thickness.

The ability to control the penetration of the cement paste into the particle-bed is crucial for the sufficient layer bonding and the successful application of SPI (see Figure 1). This requires a profound knowledge of the interplay between the rheology of the cement paste and the flow resistance of the particle-bed.

In case of a material change or a batch changeover, it is currently still necessary to carry out material- and time-consuming “trial and error” tests to prove the success of the cement paste (particle-bed) layer thickness combination selected for production. This can be very uneconomical, especially when using large printers. In order to avoid this procedure in future, prediction of the penetration depth of the cement paste by means of simple material tests and analytical models is required. A first approach of an analytical model to calculate the penetration of cement pastes into particle-beds by Pierre et al. [44] showed good results to predict a penetration of the cement paste into the particle-bed under the assumption that the penetration ends at the border to the next layer of the particle-bed. Moreover, numerical simulation appears to be an alternative for the prediction of the penetration depth [45,46].

This paper presents analytical models which are able to calculate penetration depths which are even deeper than a particle-bed layer. Furthermore, the models in this paper try to take the flow behavior during the application of the cement paste on the particle-bed into account, before the penetration starts, which enables a slight modification of a model already existing by Pierre et al. [44] (model A) to be used for penetration depths greater than the layer height (model A+). Additionally, a new approach for an analytical implementation of the pore structure of the particle-bed and of the process-parameters of the printer (applied volume as well as nozzle diameter) are considered.

Therefore, a generalized and an advanced generalized model as well as a simplified model (with easier determination of the input parameters) will be developed based on Darcy´s law [44,47,48]. The results of the analytical models will be compared to experimentally determined penetration depths which were varied by the rheological properties of the cement paste, the size of the aggregates in the particle-bed with constant packing density (flow resistance) and the humidity of the aggregates (dry and wet).

## 2. Materials and Methods

### 2.1. Selective Paste Intrusion (SPI) Method

The Selective Paste Intrusion (SPI) is able to create a link between the depositing processes with their high strengths and the particle-bed based processes with their high surface resolution combined with high durability. In SPI, only the aggregate is applied as dry material which is bound by a local intrusion of cement paste.

**Figure 3:**
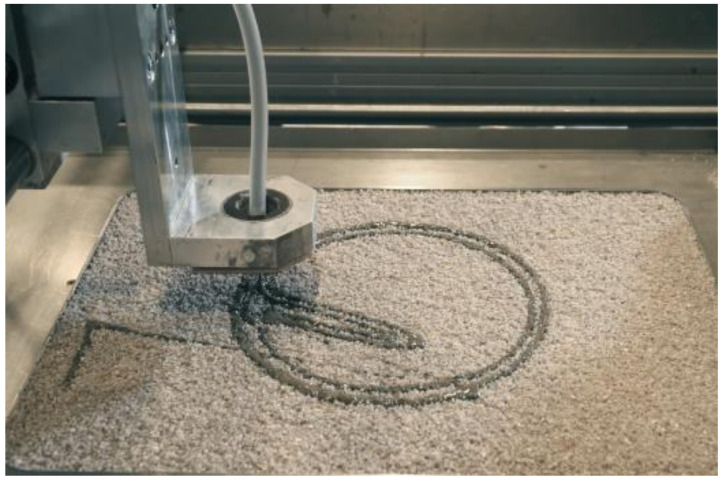
SPI printer used for the investigations [49].

The experimental setup of the printer used for the investigations in this paper consists of a conical, round nozzle with an inner diameter b_nz_ of 2.0 mm, which moves with a height h_nz_ of 15.0 mm over the particle-bed with dimensions of 0.305 m to 0.375 m by means of an x-y gantry system. The height of the building space is 0.250 m, see Figure 3.

The cement paste is pumped from a reservoir to the nozzle by a peristaltic pump controlled by a stepping motor. After the local application of the cement paste on the particle-bed, the building platform, on which the particle-bed is applied, is lowered by the set layer thickness and a new particle layer is applied (here 3.0 mm). After the printing is finished, the component is excavated from the building space.

A major advantage is the almost dust-free unpacking process and the good flowability of the unbound particles, which results in easy unpacking. The unconsolidated aggregate can be reused for a new print.

### 2.2. Materials

#### 2.2.1. Cement Paste

**Figure 4:**
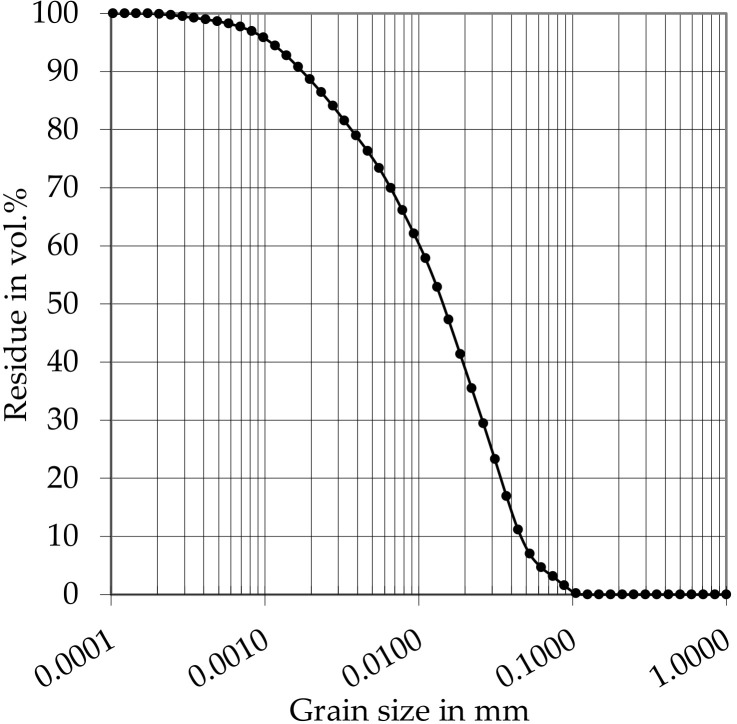
Grain size distribution of the used OPC.

An Ordinary Portland Cement (OPC, cement type: CEM I 42.5 R) was used as cement for all investigations. The grain size distribution of the cement can be taken from Figure 4. Demineralized water was utilized as mixing water. Furthermore, a polycarboxylate ether-based superplasticizer (PCE) with a solid content of 35.1 wt.%. was applied. The water content of the PCE was charged in the mixing water.

The PCE was mixed into the mixing water. The cement was placed in the mixer and the solution of water with the PCE was added over the first 30 s of the mixing process. The first mixing section lasted 90 s, followed by a 120 s pause in order to return cement adhering to the bottom of the mixing container and to the mixing tool into the mixing process. Then, the mixing process was continued for another 90 s. The device used was an intensive mixer with a star type rotor (Eirich, type R 02) at fastest speed (stage 2 of 2 for mixing tool and container). The mixing water was pre-cooled to 1.5 °C using a cryostat in order to achieve a cement paste temperature of 20 ± 1 °C.

The investigations were performed using cement pastes with different water to cement ratios (w/c-ratio 0.30, 0.35 and 0.40) as well as mini slump flows (250 mm, 300 mm, 350 mm and 400 mm). An overview of the cement pastes with the superplasticizer contents used which allows to reach the targeted mini slump flows can be found in Table 1.

The mini slump flow (measured with a Haegermann cone) correlates with the yield stress [50,51]. In addition, the viscosity (as well as the thixotropy) of the cement paste change with the w/c-ratio for the same mini slump flow. The density and the results of the rheological measurements of the cement paste can be found in Appendix A, Table A1.

#### 2.2.2. Aggregates

Three fractions of a sieved and fire-dried quartz sand from the same deposit with a grain size of 0.7–1.2 mm (medium grain size d_50_ = 1 mm), 1.0–2.2 mm (d_50_ = 1.6 mm) and 2.0–3.2 mm (d_50_ = 2.6 mm) were used.

All fractions exhibit comparable densities as well as bulk densities and thus similar porosity contents (ratio between cavity volume to total volume), see Table 2. This causes a comparable requirement of cement paste in order to fill the cavities. Assuming a same surface roughness and moisture content, the flow resistance, is thus only determined by the d_50_ or by the size of the gaps between the particles, respectively.

### 2.3. Experimental Methods

#### 2.3.1. Rheological Measurements

The yield stress and the viscosity were determined in a rotational double-plate measuring system (diameter 50 mm, gap distance 1 mm, rheometer: Anton Paar MCR 502) 900 s after addition of the mixing water. The plates had a surface roughness with a depth of 0.5 mm to prevent wall slip. The profile started with an average shear rate of 40 s^−1^ for 10 s to achieve a complete structural break up (for the given shear history). Afterwards, the measurement was carried out with 19 descending steps of 80 s^−1^ to 0.02 s^−1^ with a step duration of 6 s. Preliminary tests showed that with this profile an equilibrium (an approximately constant value of the shear stress in every step) could be achieved in the steps between 60 s^−1^ and 2.5 s^−1^ for all measured pastes. Below 2.5 s^−1^, the effect of thixotropy was already recognizable by a rising value of the shear stress.

The yield stress and the viscosity were calculated using the model of Herschel–Bulkley, which is a common model to describe the flow behavior of cement pastes [52,53,54,55] (see Equations (1) and (2)).

(1) $$\tau =\tau_{0,\mathrm{HB}}+k\cdot {\dot{\gamma }}^{n},$$

τ_0,HB_ (Pa) is the (Herschel–Bulkley) yield stress and k (Pa∙s^n^) is the consistency factor. In addition, the flow index n [-] describes a shear-thinning behavior in the range 0 < n < 1 and a shear-thickening behavior in the range 1 < n < ∞. If n = 1, it becomes a Bingham fluid.

The viscosity of Herschel–Bulkley is always given as a function of shear rate. Consequently, the shear rate-dependent, (Herschel–Bulkley) viscosity η($\dot{\gamma }$) (Pa∙s) is calculated according to Equation (2).

(2) $$\eta (\dot{\gamma })=\frac{\tau_{0,\mathrm{HB}}}{\dot{\gamma }}+k\cdot {\dot{\gamma }}^{n-1}\;,$$

#### 2.3.2. Penetration Tests

**Figure 5:**
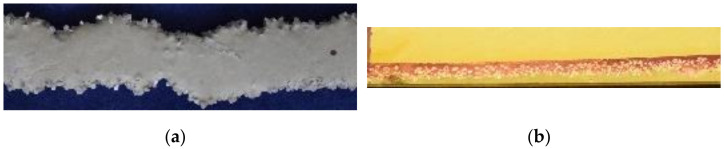
0.10 m long section of a strand with d_50_ = 0.001 m; (**a**) View from top; (**b**) Horizontal view on the lengthwise cross section of a resin-coated strand which is sprayed with phenolphthalein.

**Figure 6:**
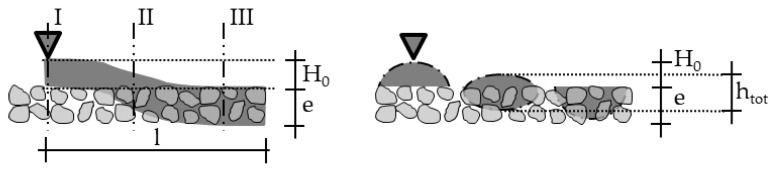
Measurement of the penetration depth e and the total height h_tot_ of the strands; I: Not penetrated cement paste with height H_0_; II: Partly penetrated cement paste; III: Completely penetrated cement paste.

For the measurement of the penetration depth noted e, two strands of cement paste with a length of each 0.25 m per particle fraction were applied (see Figure 5 and Figure 6). Thus, the cement paste could penetrate the particle-bed without reaching the bottom of the building space.

In order to quantify the effect of the process parameters, the penetration depth was investigated for constant cement paste properties (w/c-ratio 0.3 and mini slump flow of 300 mm) by changing (a) the velocity of the nozzle (by gantry movement speed) at the same volume output per length and by changing (b) the volume output per length at constant gantry movement speed (see Appendix B, Table A2).

All tests were carried out on both dry and pre-wetted (wet) aggregates. Pierre et al. have recently shown that aggregates water content affects the penetration depth [45]. For pre-wetting, aggregates were stored under water for at least 12 h and removed from the water before printing. The wet aggregate surface was dabbed with paper towels to quantify the dehydration effect on the cement paste by a dry aggregate.

The hardened strands were poured into epoxy resin, sawed apart lengthwise in the middle and sprayed with phenolphthalein in order to clearly distinguish the cement matrix from the aggregates. Then, the penetration depth e of the strands was measured every 0.01 m which results in 50 values for the penetration depth per series (see Figure 5 and Figure 6).

## 3. Penetration Models

### 3.1. Concept of Model Development

A first approach of an analytical model to calculate the penetration of cement pastes into particle-beds was proposed by Pierre et al. [44] (model A). Based on Darcy´s law and the Green and Ampt equation [44,47,48], model A showed good results to predict a penetration of the cement paste into the particle-bed under the assumption that the penetration ends at the border to the next layer of the particle-bed, which makes it impossible to generally use it in the SPI-printing process.

Therefore, this paper presents analytical models which are able to calculate penetration depths which are even deeper than a particle-bed layer. Furthermore, the models in this paper take the flow behavior during the application of the cement paste on the particle-bed into account, before the penetration starts, which enables model A also to be used for penetration depths greater than the layer height (model A+). Additionally, a new approach for an analytical implementation of the pore structure of the particle-bed and of the process-parameters (applied volume as well as nozzle diameter) are inserted directly in the model or indirectly by the calculation of the spread characteristic of the strands.

Thus, a generalized model (model C), an advanced generalized model (model D) as well as a simplified model (model B/B+), with easier determination of the input parameters, are developed based on Darcy´s law [44,47,48]. The results of the analytical models will be compared to experimentally determined penetration depths which were varied by the rheological properties of the cement paste, the size of the aggregates in the particle-bed (flow resistance) and the humidity of the aggregates (dry and wet).

**Figure 7:**
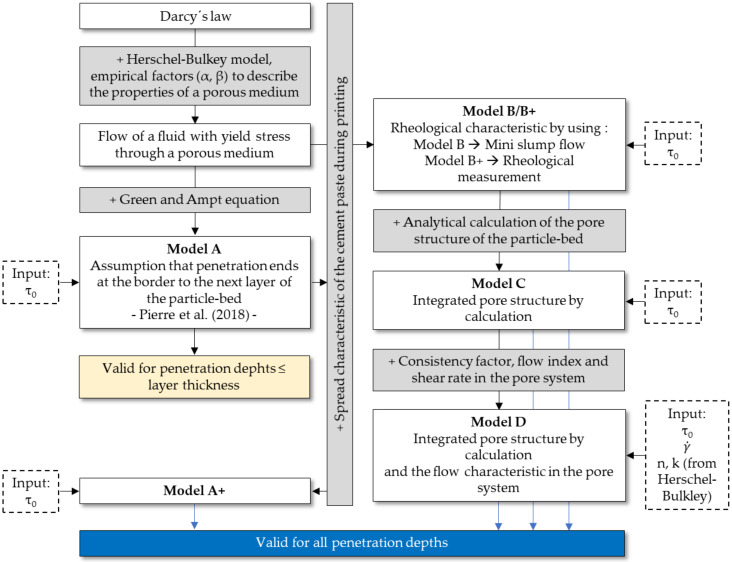
Model development for the analytical calculation of the penetration depth.

Figure 7 illustrates the path of the model´s development. Model B describes the penetration behavior only with the help of the mini slump flow value of the cement paste, whereas model B+ uses the yield stress estimated from rheological measurements for the calculation of the penetration depth.

Models C and D use a novel approach to calculate the factors α and β, which are usually values determined by experiments to describe the pore structure [47,48]. This is done by an assumption of an effective capillary pore system. Model C describes a simpler variant, since only the yield stress is used as a rheological input parameter. In model D, the flow behavior in the porous medium is extended by the shear rate occurring in the pores $\dot{\gamma }$ and the Herschel–Bulkley parameters n and k. This can improve the results especially for very flowable pastes.

All presented models assume a pressureless deposition of the cement paste on the particle-bed (laminar flow) and thus only the dead weight of the paste (hydrostatic pressure) as driving force of the penetration against the flow resistance of the particle-bed.

If additional pressure is applied via the nozzles, it has to be considered in addition to the hydrostatic pressure. The applied pressure can furthermore modify the flow regime itself and thus affect the reliability of the whole penetration process. For very high pressures, the type of flow could change from laminar to turbulent and the resulting loss of pressure could make it necessary to apply, e.g., the Forchheimer equation [56,57,58]. However, this is not part of the presented models.

The presented models assume that the penetration of the cement paste is considerably shorter than the setting and hardening time. Therefore, the penetration must be at least 10 times faster than the setting/hardening kinetics. This is also valid for the thixotropy of the cement pastes used in this investigation, which is neglected in this approach.

The change of process-technological aspects like a) the velocity of the nozzle (by gantry movement speed) at the same volume output changing and b) the volume output at constant gantry movement speed (see Section 2.3.2 and Appendix B, Table A2) showed no significant effect on the penetration depths in the selected test setup; see also [34]. Therefore, the velocity of the nozzle as an influencing factor is neglected for the models presented in this paper. However, the deposited volume of material could have an effect and is nevertheless implemented.

### 3.2. Theoretical Background of the Models

Many studies of a flow through porous media of non-Newtonian fluids concentrate mainly on the description of viscous power laws fluids without having a yield stress [59,60,61,62,63,64]. However, especially when considering the flow behavior of concretes or cement pastes, e.g., in the field of oil well cementing, soil injection or the simulation of the flow of Self Compacting Concrete (SCC) through rebars [65,66,67], the consideration of the yield stress is decisive.

Chevalier et al. [47,48] combined Darcy’s law with the Herschel–Bulkley model assuming spherical particles in the particle-bed in order to model the flow of a fluid with a yield stress through a porous medium, see Equation (3).

(3) $$D\nabla p=\alpha \cdot \tau_{0}+\beta \cdot k\cdot {\left(\frac{v}{D}\right)}^{n},$$

In which D (m) is an undefined unit of length that characterizes the porous medium and ∇p (Pa/m) is the pressure loss over this unit of length. α (here = 5.5) and β (here = 85) are two unknown parameters which must be determined experimentally by flow measurements.

α depends on the size ratio of the particles in the fluid and the widest path between the particles of the particle-bed. β describes the flow resistance of the entire particle-bed depending on the pore size distribution and structure. β also considers the permeability K (m^2^) of the particle-bed. Assuming a Newtonian fluid (τ_0_ = 0 and n = 1), the following relationship can be obtained using Darcy’s law and the Kozeny–Carman equation [44,68], see Equation (4).

(4) $$\nabla p=\beta \cdot k\cdot \frac{v}{D^{2}}=k\cdot \frac{v}{K}$$

The ratio of the flow velocity of the fluid v (m/s)/D (m) is equivalent to the shear rate $\dot{\gamma }$ (s^−1^) occurring between the particles in the porous medium.

In Equation (3), the first term including the yield stress describes the mobilization of the fluid in the widest path through the porous medium. As the shear rate increases, the fluid also flows in finer pores (second term). Thus, the ratio of the pressure drop increases with the activation of smaller pores [47].

However, when using this model, it should be noted that the particles in the fluid should be much smaller than the particles of the particle-bed.

However, the literature [47,56,63,69] presents very large ranges of α (0.98–5.5) and β (1.23–102.7) and mutual dependencies. Therefore, the coefficients should always be determined experimentally. However, in the case of cement-based suspensions, this is very difficult due to the time-dependent flow properties. Consequently, this paper shows a new assumption for the calculation of these coefficients.

### 3.3. Spread Characteristic of the Cement Paste During Printing

**Figure 8:**
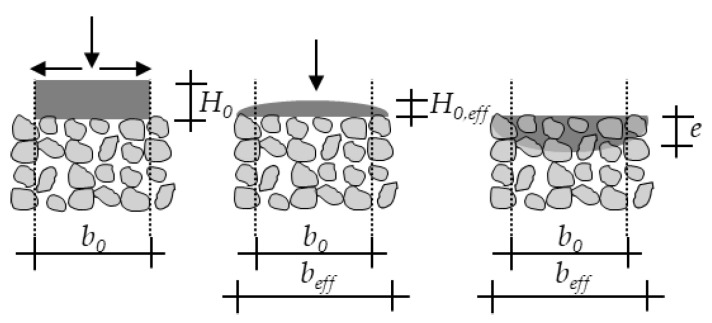
Spread characteristic of the cement paste; H_0_: Initial height of not penetrated cement paste; b_0_: Initial width of the not penetrated cement paste; b_eff_: width of the cement paste immediately before penetration; H_0,eff_: effective height of the cement paste, e: penetration depth.

**Figure 9:**
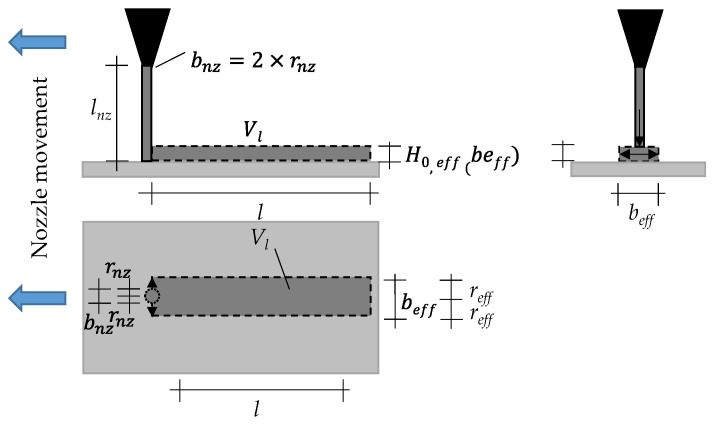
Application of the cement paste during printing process; b_nz_: diameter of the nozzle; r_nz_: Radius of the nozzle; l_nz_: height of the nozzle; H_0,eff_: effective height of the cement paste (strand); b_eff_: width of the cement paste (strand) immediately before penetration, r_eff_: Halved width (radius) of the cement paste (strand) immediately before penetration; l: length of the strand; V_l_: Applicated volume.

The models of this paper assume that during the printing process the cement paste does not only flow vertically from the nozzle into the particle-bed, but spreads also horizontally on the particle-bed before it penetrates (see Figure 8 and Figure 9). Application of the cement paste during printing process).

The applied cement paste strand with an initial height H_0_ (m) and width b_0_ (m) will spread aside to a width b_eff_ (m) and a related effective height H_0,eff_ (m) which determines the effective hydrostatic pressure as driving force for the penetration.

By using fine nozzles and thus thin strands of cement paste comparing to the applied length l (m), we can assume a rectangular cross section of the strand. Following [50,70,71] and under the condition that the cement paste is not penetrating the particle-bed before the strand has spread; we can further assume that the final spread b_eff_ is depending on the yield stress of the cement paste. Thus, the yield stress determines the final height H_0_._eff_ (and therefore the effective hydrostatic pressure) of the strand.

Therefore, Equation (5) describes the final condition (h = H_eff,0_) of the spread before penetrating the particle-bed.

(5) $$\tau_{o}=\rho_{p}\cdot g\cdot h\cdot \frac{\mathrm{dh}}{\mathrm{db}}=\rho_{p}\cdot g\cdot \int_{b_{\mathrm{nz}}}^{b_{\mathrm{eff}}}h\cdot \mathrm{db}\;=\rho_{p}\cdot g\cdot \frac{h^{2}}{2\cdot l_{\mathrm{nz}}}\cdot \left(\frac{1}{b_{\mathrm{eff}}}-\frac{1}{b_{\mathrm{nz}}}\right)\;,$$

Here, τ_0_ (Pa) is the yield stress, ρ_p_ (kg/m^3^) is the density of the cement paste, g (m/s^2^) is the gravity and b_nz_ (m) the diameter of the nozzle.

Thus, for h = H_0_._eff_, the equation turns to Equation (6).

(6) $$H_{0,\mathrm{eff}}=\sqrt{\frac{\tau_{0}\cdot 2\cdot \left(b_{\mathrm{eff}}-b_{\mathrm{nz}}\right)}{\rho_{p}\cdot g}}$$

However, the effective width b_eff_ of the strand is still not known. Again, under assumption of a rectangular cross section and already achieved final spread b_eff_ (b_nz_ = 0), the applied volume V_l_ (m^3^) can be calculated with the length l (m) of the strand and H_0,eff_ using Equation (7).

(7) $$V_{l}=b_{\mathrm{eff}}\cdot l\cdot H_{0,\mathrm{eff}}\left(b_{\mathrm{eff}}\right)=\sqrt{\frac{\tau_{0}\cdot 2\cdot b_{\mathrm{eff}}}{\rho_{p}\cdot g}}\cdot l\cdot b_{\mathrm{eff}}$$

This leads to Equation (8) for calculation of b_eff_.

(8) $$b_{\mathrm{eff}}=\sqrt[{3}]{\frac{V_{l}^{2}\cdot \rho_{p}\cdot g}{l^{2}\cdot \tau_{0}\cdot 2}}\;,$$

Consequently, the yield stress τ_0_ can be calculated following equation (9), when b_eff_ is known.

(9) $$\tau_{0}=\frac{V_{l}^{2}\cdot \rho_{L}\cdot g}{2\cdot l^{2}\cdot b_{\mathrm{eff}}^{3}}\;,$$

However, the final height H_0,eff_ can also simplified be approximated by the final height h(R) of the cement paste after the mini slump flow test following [50,51], which was used in [34] (see Appendix C).

### 3.4. Modification of Model A to Model A+

A detailed derivation of model A (see Equation (10)), can be found in [44].

(10) $$e_{A}=\frac{\rho_{L}\cdot g\cdot d_{50}\cdot \left(1-\varphi \right)\cdot h_{\mathrm{lay}}}{\alpha \cdot \tau_{0}-\rho_{L}\cdot g\cdot d_{50}\cdot \varphi +\frac{\varphi }{\left(1-\varphi \right)}\cdot 6\cdot \tau_{0}\cdot \kappa },$$

Here, φ is the solid fraction of the porous medium, h_lay_ (m) the layer height of the applied particles and κ the Janssen parameter [72].

In order to make the calculation also valid for penetration depths greater than the layer height h_lay_, model A has to become independent to this parameter (see [34]). Therefore, all parts of Equation (10) which describe the height of the cement paste depending on h_lay_ have to be replaced by Equation (6) which leads to Equation (11).

(11) $$e_{A+}=\frac{\rho_{L}\cdot g\cdot d_{50}\cdot H_{0,\mathrm{eff}}}{\alpha \cdot \tau_{0}+\frac{\varphi }{\left(1-\varphi \right)}\cdot 6\cdot \tau_{0}\cdot \kappa },$$

### 3.5. Theoretical Background of Model B/B+

For a first and simple approach in model B/B+ the β-term associated to the viscosity effect will be neglected. Thus, Equation (3) simplifies to Equation (10).

(12) $$D\nabla p=\alpha \cdot \tau_{0}\;,$$

Consequently, as limiting boundary condition for a penetration of the cement paste, the yield stress must firstly be overcome [44] (see Equation (12)).

(13) $$\nabla p>\frac{\alpha \cdot \tau_{0}}{D}{\;\mathrm{or}\;\tau }_{0}<\frac{D\cdot \nabla p}{\alpha }\;,$$

For model B/B+, it is assumed that the medium particle diameter d_50_ (m) can be used for D as the determining factor for the flow resistance of the particle-bed. According to Chevalier et al. [47], coefficient α is set to 5.5. Furthermore, the effective hydrostatic pressure decreases over time. This is taken into account by a linear regression [73], which can be expressed by dividing the effective hydrostatic pressure H_0,eff_ from Equation (6) by 2.

Following this assumption and that the hydrostatic pressure turns to zero after reaching the final penetration depth e (∇p = ρ_p_ ∙ g H_0,eff/_e), Equation (12) turns into model B/B+ in Equation (14).

(14) $$e_{B/B+}=\frac{\rho_{P}\cdot g\cdot \frac{H_{0,\mathrm{eff}}}{2}\cdot d_{50}}{5.5\cdot \tau_{0}}\;,$$

The difference between models B and B+ is only the measurement of the yield stress. Model B does not need a rheological measurement by a rheometer but uses the assumption of Roussel [50] to determine the yield stress only by the slump flow value. In contrast, model B+ utilizes the results of the rheometric measurements following the model of Herschel–Bulkley, which probably enables more accurate results.

### 3.6. Theoretical Background of Model C and D

Since model B/B+ use for α values from literature or difficult to determine experimental values, models C and D calculate the values for α and β according to the pore structure.

Therefore, we assume that the porous medium of the particle-bed can be described by a capillary pore structure between the particles. Consequently, the determinant factor for the flow resistance turns to the effective pore diameter d_eff_ (m) (=D).

Chevalier et al. [47] describe that α defines the penetration ability of a fluid which depends on the widest path between the particles in the particle-bed or on the porosity of the particle-bed ε, respectively. Consequently, α can be assumed according to Equation (15).

(15) $$\alpha ∼\frac{1}{\varepsilon }\;,$$

In combination with Equation (15), Equation (12) or (14), respectively, turn to model C in Equation (16).

(16) $$e_{C}=\frac{\rho_{P}\cdot g\cdot \frac{H_{0,\mathrm{eff}}}{2}\cdot d_{\mathrm{eff}}\cdot \varepsilon }{\tau_{0}}\;,$$

In contrast, β describes the flow resistance of the entire particle-bed as a function of pore size distribution and structure. Thus, β designates the mobilization of the fluid in the pores of the particle-bed (porosity ε) depending on the limiting yield stress τ_0,lim_ (Pa) (depending on the pore size distribution and structure) as well as the actual yield stress of the fluid τ_0_. This results in the following assumption for factor β, see Equation (17).

(17) $$\beta ∼\frac{1}{\varepsilon \cdot \frac{\tau_{0}}{\tau_{0,\lim}}}\;,$$

Implementing Equations (15) and (17) in Equation (3) under the further assumption of the boundary conditions of model C gives model D in Equation (18).

(18) $$e_{D}=\frac{\rho_{P}\cdot g\cdot \frac{H_{0,\mathrm{eff}}}{2}\cdot d_{\mathrm{eff}}\cdot \varepsilon }{\tau_{0}+\frac{\tau_{0,\lim}}{\tau_{0}}\cdot k\cdot {\;\dot{\gamma }\;}^{n}}\;,$$

### 3.7. Determination of the Effective Pore Structure and Shear Rate in the Particle-Bed

**Figure 10:**
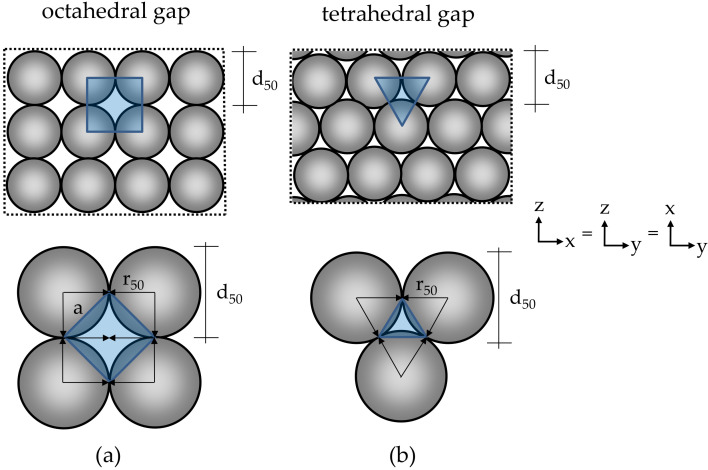
Sphere packing with (**a**) octahedral and (**b**) tetrahedral gaps; Top: Cross section of sphere packing with gaps; Bottom: Detailed view of a gap between the particles (gray); a: Side length of the triangle; d_50_: Medium grain size (diameter); r_50_: Halved medium grain size (radius).

Assuming that the particle-bed consists of a dense, hexagonally arranged packing (hexagonally densest packing or cubically densest packing), tetrahedral or octahedral gaps occur between the particles [63,74]. If the tetrahedral gaps are approximated as an equilateral triangle and the octahedral gap as a square or two isosceles triangles, the area and circumference can be determined (see Figure 10).

If the area of the octahedral gap A_octa_ (m^2^) is derived from two isosceles triangles (see Equation (19)), the circumference of the octahedral gap U_octa_ (m) can be determined in Equation (20).

(19) $$A_{\mathrm{octa}}=2\cdot \left(\frac{1}{2}\cdot \left(2\cdot r_{50}\right)\cdot r_{50}\right)=2\cdot r_{50}^{2}=\frac{1}{4}\cdot \left(2\cdot r_{50}\right)\cdot \sqrt{4\cdot a^{2}-{\left(2\cdot r_{50}\right)}^{2}}\;,$$

(20) $$U_{\mathrm{octa}}=4\cdot \sqrt{\frac{{\left(\frac{4\cdot A_{\mathrm{octa}}}{2\cdot r_{50}}\right)}^{2}+{\left(2\cdot r_{50}\right)}^{2}}{2}}\;,$$

The area of the equilateral triangle of the tetrahedral gap A_tetra_ (m^2^) can be determined according to Equation (21). The circumference of the tetrahedral gap U_tetra_ (m) is calculated according to Equation (22).

(21) $$A_{\mathrm{tetra}}=\frac{\sqrt{3}}{4}\cdot r_{50}^{2}\;,$$

(22) $$U_{\mathrm{tetra}}=3\cdot r_{50}\;,$$

If the area and circumference of the pore is known, an equivalent effective pore radius r_eff_ [m] of a round capillary can be calculated according to Lopez et al. [63] in Equation (23).

(23) $$r_{\mathrm{eff}}={\left(\frac{8\cdot G}{\pi }\right)}^{\frac{1}{4}}\;,$$

G can be calculated using Poiseuille’s law according to Equation (24) and form factor χ [75], see Table 3.

(24) $$G=\chi \cdot A_{\mathrm{octa}/\mathrm{tetra}}^{2}\cdot \frac{A_{\mathrm{octa}/\mathrm{tetra}}}{U_{\mathrm{octa}/\mathrm{tetra}}^{2}}\;,$$

With the effective equivalent radius r_eff_ an equivalent capillary pore system can now be approximated. In a pressureless capillary pore, the shear rate $\dot{\gamma }$ can be given as a function of the effective pore radius r_eff_ and the shear rate-dependent viscosity η [73] (see Equation (25)).

(25) $$\;\dot{\gamma }\;=\frac{\rho_{P}\cdot g\cdot H_{0,\mathrm{eff}}\cdot r_{\mathrm{eff}}}{2\cdot L_{\mathrm{eff}}\cdot \eta }\;,$$

L_eff_ (m) describes an equivalent effective limiting length of a capillary with the radius r_eff_ and can be determined among others following [63,76,77,78,79,80] according to Equation (26).

(26) $$\mathrm{Leff}=\sqrt{K\cdot \varepsilon }\;,$$

**Figure 11:**
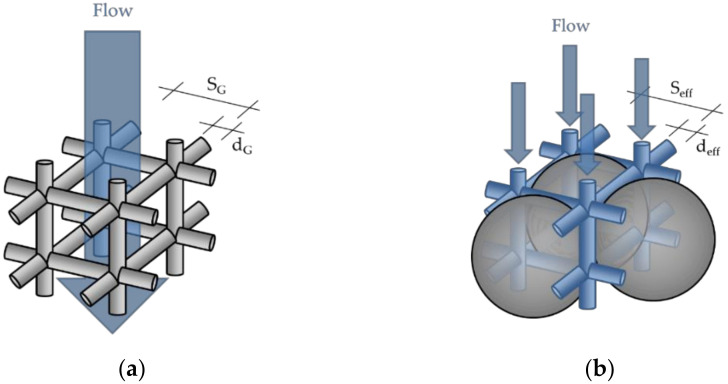
(**a**) Simple cubic 3D arrangement of cells in a porous medium; (**b**) 3D arrangement of the capillaries between the particles; S_G_: Centric distance of the non-permeable part of the lattice; d_G_: Diameter of the lattice bars; S_eff_: Centric distance of the permeable capillaries; d_eff_: Diameter of the permeable capillaries.

There, K (m^2^/Darcy) is the absolute permeability coefficient. This coefficient can be calculated by analogies for the simulation of flow movements in porous media. Tamayol et al. [69,81] describe the pore structure of a three-dimensional porous medium simplified with a three-dimensional cubic configuration of a lattice with a flow resistance that can be expressed in a lattice permeability coefficient K_G_ [m^2^] (see Equation (27) and Figure 11a.

(27) $$\frac{K_{G}}{d_{G}^{2}}=0.08\frac{{\left(S_{G}-d_{G}\right)}^{4}}{S_{G}^{2}\cdot d_{G}^{2}\cdot \varepsilon^{0,3}}\;\mathrm{or}\;K_{G}=0.08\frac{{\left(S_{G}-d_{G}\right)}^{4}}{S_{G}^{2}\cdot \varepsilon^{0,3}}\;,$$

The constant 0.08 describes the arrangement of the 3D lattice for a wide range of porosities and was found by Tamayol and Bahrami [69] by comparison with numerical data from Higdon and Ford [82]. S_G_ [m] is the centric distance of the non-permeable part of the lattice and d_G_ [m] the diameter of the lattice bars. The parameters S_G_ and d_G_ in three-dimensional structures can be expressed by the relationship in Equation (28) [69,82].

(28) $$\varphi =\frac{3\cdot \pi \cdot d_{G}^{2}}{4\cdot S_{G}^{2}}-\sqrt{2}\cdot \frac{d_{G}^{3}}{S_{G}^{3}}\;,$$

In this case, S_G_ (m) and d_G_ (m) are unknown parameters and φ is the solid ratio.

**Figure 12:**
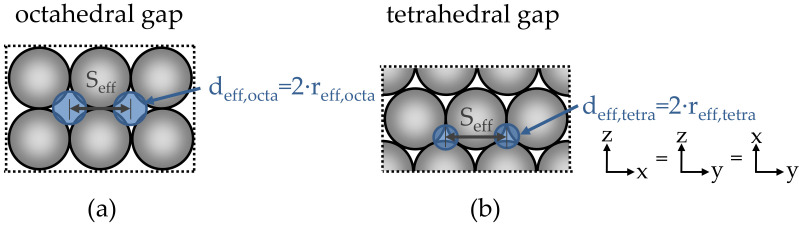
3D capillary system of the particle bed with (**a**) octahedral and (**b**) tetrahedral gaps; d_eff_: Diameter of the permeable capillaries; r_eff_: Radius of the permeable capillaries.

However, if we now assume that the lattice system itself can be flowed through, and that the sections between the lattice no longer have porosity, the three-dimensional capillary system of the particle-bed results (see Figure 11b and Figure 12).

Now the porosity of the particle bed K_3D_ (m^2^) for octahedral d_eff_,_octa_ (m) or tetrahedral gaps d_eff,tetra_ (m) can be represented according to Equations (29) and (30).

(29) $$K_{3D}=0.08\frac{{\left(S_{\mathrm{eff}}-d_{\mathrm{eff}}\right)}^{4}}{S_{\mathrm{eff}}^{2}\cdot \varphi^{0,3}}\;,$$

(30) $$\varepsilon =\frac{3\cdot \pi \cdot d_{\mathrm{eff}}^{2}}{4\cdot S_{\mathrm{eff}}^{2}}-\sqrt{2}\cdot \frac{d_{\mathrm{eff}}^{3}}{S_{\mathrm{eff}}^{3}}\;,$$

Then, the effective capillary length L_eff_ can be determined by combining Equations (26), (29) and (30).

According to Hagen–Poiseuille, a limiting boundary condition for the penetration of the cement paste can now be given in this capillary system analogous to Equation (13) in combination with the largest effective radius of the pore system r_eff,octa_ (see Equation (31)).

(31) $$\tau_{0,\lim,C,D}=\frac{\rho_{P}\cdot g\cdot H_{0,\mathrm{eff}}\cdot r_{\mathrm{eff},\mathrm{octa}}}{2\cdot L_{\mathrm{eff}}}\;,$$

Since the dynamic viscosity η is dependent on the shear rate $\dot{\gamma }$, the shear rate according to Equation (25) still cannot be calculated despite L_eff_ being known. However, the limiting yield stress τ_0,lim,C,D_ according to Equation (31) can be used, which characterizes the transition of the fluid into the flow state. By combining Equations (2), (25) and (31), Equation (32) can be derived for the calculation of the shear rate $\dot{\gamma }$ in the pores of the particle-bed.

(32) $$\dot{\gamma }=\frac{\tau_{0,\lim}}{\left(\frac{\tau_{0}}{\dot{\gamma }}+\;k\cdot {\dot{\gamma }}^{n-1}\right)}\;\dot{\gamma }=\sqrt[{n}]{\frac{\tau_{0,\lim}-\tau_{0}}{\;k}}\;\mathrm{for}\;{\;\tau }_{0,\lim}>\tau_{0}\;\mathrm{and}\;\;\dot{\gamma }\;=0\;\mathrm{for}\;\tau_{0,\lim}\leq \tau_{0}\;,$$

## 4. Results and Discussion

**Figure 13:**
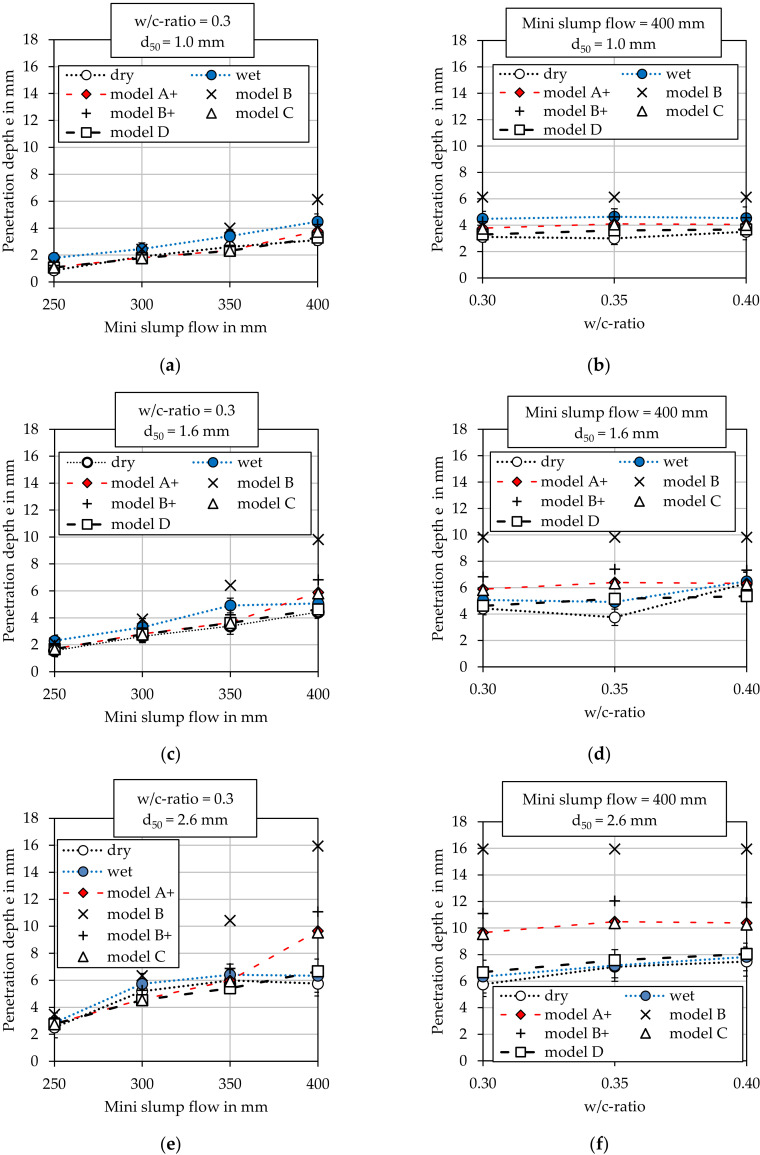
Measured and calculated penetration depths e (model C and D using tetrahedral gaps) depending on the mini slump flow (**a**,**c**,**e**) and on the w/c-ratio (**b**,**d**,**f**) as well as on the aggregate size (d_50_) and humidity of the aggregate (wet and dry).

Figure 13 shows the main results of the model validation by comparison with real measured penetration depths e depending on the mini slump flow in (a), (c), (e) with a constant w/c-ratio of 0.3 and depending on the w/c-ratio in (b), (d), (f) with a constant mini slump flow of 400 mm.

The measured penetration depths (white and blue circles) increase with increasing slump flow (decreasing yield stress) and increase slightly with increasing w/c-ratio (decreasing viscosity). Thus, the yield stress exhibits a dominant role in comparison to the viscosity for the penetration of the cement paste. Furthermore, the penetration depth e increases with an increasing d_50_ (decreasing flow resistance of the particle-bed) and with increasing humidity of the particle-bed (circles in white = dry and blue = wet). These observations correspond to the results in [25,26,28,31,32,44].

Model A+ exhibits a good correlation to the penetration depths up to a mini slump flow of 350 mm with a deviation of ≤0.6 mm for dry aggregates and ≤1.3 mm for wet aggregates. However, the deviation for a slump flow of 400 mm increases to ≤3.9 mm for dry and ≤3.3 mm for wet aggregates.

Model B show only small deviations from the measured penetration depths when considering the dry aggregates in the range of a mini slump flow from 250 mm to 300 mm. For a low yield stress (mini slump flow 350 mm and 400 mm), the deviation of model B strongly increases with increasing particle size d_50_ (decreasing flow resistance of the particle-bed). Thus, model B overestimates the penetration depths with up to 10.2 mm (approx. +270%). The results show furthermore, that a determination of the rheolocal properties (model B+) leads to better results than using an approximation of the yield stress by the mini slump flow due to the shear thickening flow behavior of the cement paste (see results in Appendix A, Table A1).

The tendency to exhibit an increased deviation with decreasing yield stress and increasing d_50_ was also shown in Pierre et al. [44] for model A and model A+. This effect could be caused by neglecting the β-term of Equation (3). Therefore, the effect of a shear-thinning or shear-thickening flow behavior (see results in Appendix A, Table A1) cannot be reproduced in Model A and Model B/B+, although its effect increases in a porous medium with decreasing flow resistance.

Model C shows only small absolute deviations of ≤1.3 mm to the measured penetration depths for the wet aggregate up to a mini slump flow of 350 mm. However, model C shows a better agreement with the penetration depths using dry aggregate with absolute deviations of ≤0.7 mm for the same mini slump flow range. Furthermore, considering the effective pore structure in the α-term leads to better results compared to model B/B+. Comparing the results for model A+ and C, the calculated penetrations depths are almost congruent, though the approach of both models is different. However, both models seem to be able to describe the structure of the porous medium quite accurately. However, neglecting the β-term also leads to relatively high deviations for cement pastes with low yield stress (mini slump flow 400 mm) for model A+ and model C.

Model D exhibits only slight deviations of ≤1.4 mm for dry and ≤1.3 mm for wet aggregates for all penetration measurements. By applying the β-term from Equation (3), model D succeeds in eliminating the major deviations even for the flowable cement pastes with 400 mm mini slump flow.

Although the penetration depths continue to increase due to the increasing pressure gradient (τ_0,lim_/τ_0_), they are probably simultaneously reduced by the internal friction of the cement paste (respectively the dynamic viscosity, expressed by k∙ γ ˙. ^n^) and the friction on the particle surfaces. In addition, the flow through the particle-bed strongly depends on the prevailing pressure gradient and the shear rate in the pores. Due to the friction loss, the pressure gradient decreases rapidly and the flow stops more quickly as the pore size decreases. Thus, the penetration depths are additionally reduced. This effect increases with increasing ratio τ_0,lim_/τ_0_.

The results for model C and D in Figure 13 were calculated using tetrahedral gaps. The assumption of octahedral gaps showed less accurate results for the penetration depth which can be explained by the shape and bulk density of the used aggregates (see Appendix D).

The velocity of the nozzle as well as the applied volume of the cement paste (see Section 2.3.2 and Appendix B, Table A2) showed no significant influence on the penetration depth for the used printer set up (see Appendix B). However, especially higher amounts of cement paste per length could have an effect on the penetration depth.

The investigations show that model D, in contrast to the other presented models, is capable of validly calculating the penetration of the cement paste across all investigated properties of the cement pastes and particle-bed by using a pore system with tetrahedral gaps. An additional discussion of the theoretical background of the models can be found in [34,44,45,46].

**Figure 14:**
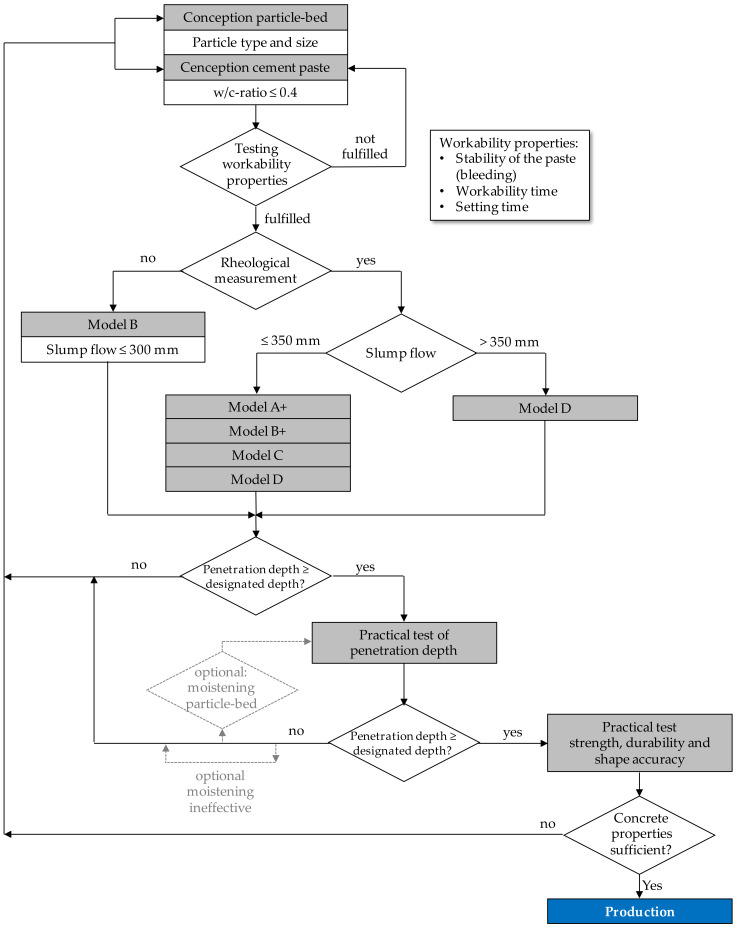
Recommended guidelines for a practical application of the SPI (valid and verified for the given boundary conditions of the paper).

Finally, the following procedure is recommended as a guideline for a successful application of SPI in practice (see Figure 14).

## 5. Conclusions and Outlook

In order to prevent effortful “trial and error” tests to validate new material combinations in the SPI printing process, we presented four models to calculate the penetration depth of a cement paste in a particle-bed based on material and process technological parameters. All models showed good results for cement pastes up to medium yield stress (mini slump flow of 300 mm to 350 mm). However, model C and D use a new approach to calculate the pore structure of the particle-bed. Model D achieved the best correlation to the real penetration measurements, even for very fluid cement paste with low yield stress (mini slump flow of 400 mm) for which viscous effects cannot be neglected.

However, the models should also be validated for other printer setups (e.g., other nozzle diameters or distances to the particle-bed). Furthermore, an expansion of the models for turbulent flow conditions could be interesting as soon as the cement paste is jetted out of the nozzle and is not deposited pressureless like for the investigations in this paper.

## Figures and Tables

**Table 1:** Investigated cement pastes with superplasticizer contents in wt.%.

w/c-Ratio	Mini Slump Flow in mm			
	250	300	350	400
0.30	0.600 wt.%	0.648 wt.%	0.695 wt.%	0.720 wt.%
0.35	-	-	-	0.560 wt.%
0.40	-	-	-	0.530 wt.%

**Table 2:** Material properties of the used aggregate.

Minimum Grain Size	Maximum Grain Size	Medium Grain Size d_50_	Density ρ_P_	Bulk Density ρ_P,B_	Porosity ε
in mm	in mm	in mm	in kg/m^3^	in kg/m^3^	*-*
0.7	1.2	1.0	2645	1414	0.465
1.0	2.2	1.6	2643	1447	0.453
2.0	3.2	2.6	2642	1434	0.457

**Table 3:** Values of the form factor χ for different cross sections of the pore according to [75].

χ	Shape of the Cross Section of the Pore
*-*	*-*
0.5	Circular shape
0.6	Equilateral triangle
0.5623	Square

**Figure A1:**
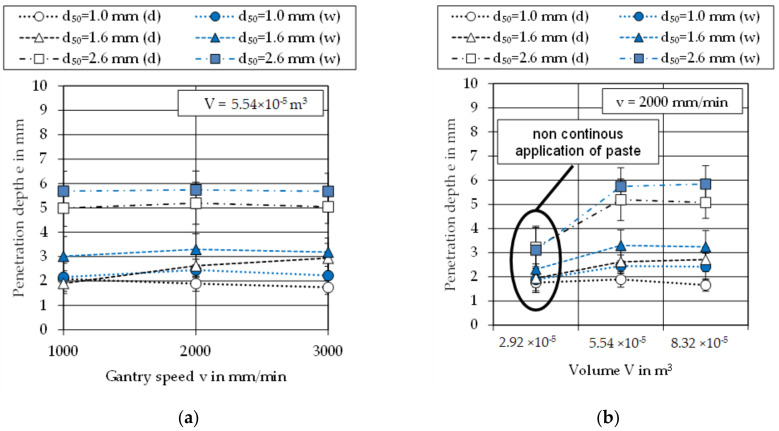
(**a**) Penetration depth e depending on the gantry speed; (**b**) Penetration depth e depending on the applied cement paste volume per length.

**Figure A2:**
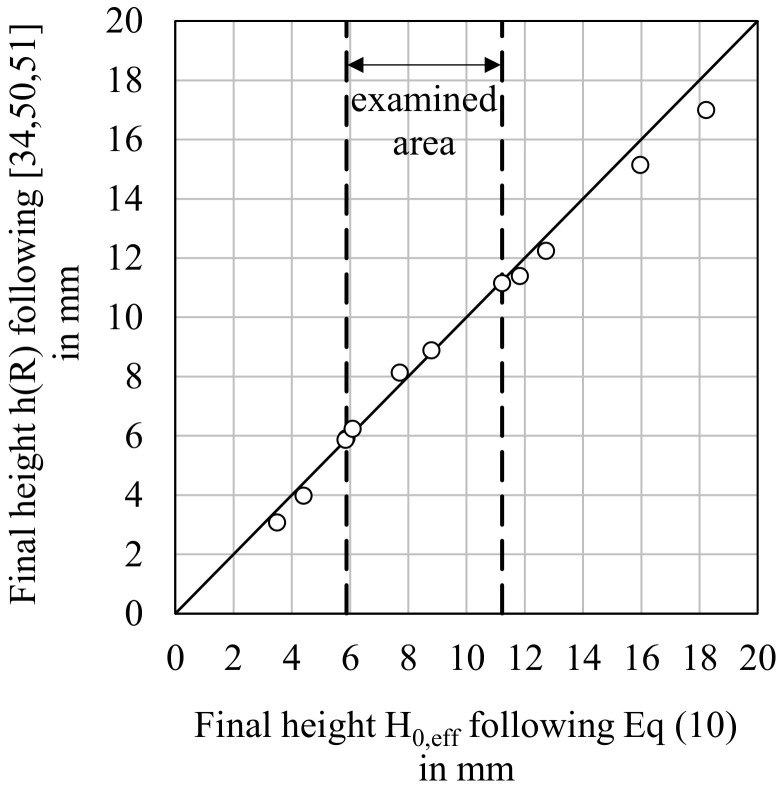
Comparison of the final height of the cement paste following Equation (10) and the final height of the cement paste h(R) after a mini slump flow following [50,51] used in [34].

**Figure A3:**
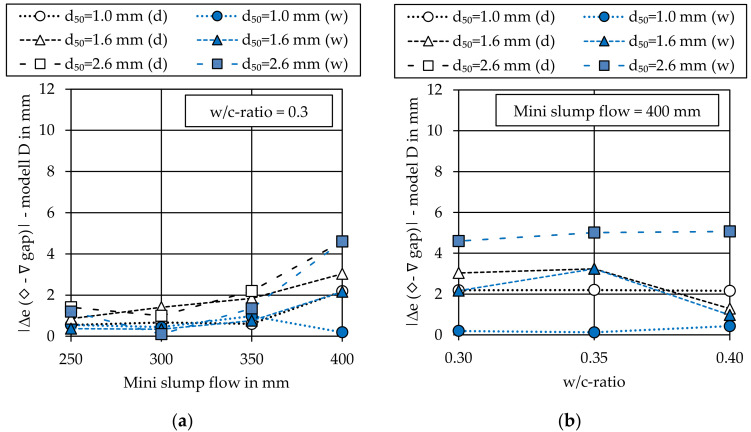
Deviation between the calculated penetration depths using octahedral gaps (◇) and tetrahedral gaps (∇) to the real measured penetration depths depending on (**a**) the mini slump flow and (**b**) the w/c-ratio.

**Figure A4:**
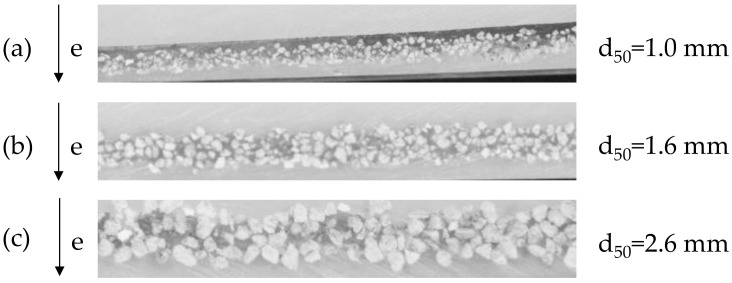
Cross section through three samples with (**a**) fine, (**b**) medium and (**c**) coarse grain size d_50_ vertically penetrated with cement paste (dark grey areas); e: direction of penetration.

## Data Availability

Data sharing is not applicable to this article.

## Appendix A

The density of the fresh cement paste was determined volumetrically according to [83]. The rheological properties were calculated according to Heschel–Bulkley. The results can be found in Table A1.

**Table A1:** Results of the density and rheological measurements according to Herschel–Bulkley.

w/c-Ratio	Mini Slump Flow	Density ρ_P_	Yield Stress τ_0_	Consistency k	Flow Index n
-	in mm	in kg/m^3^	in Pa	in Pa∙s^n^	-
0.40	400	1926	2.2	0.03	1.18
0.35	400	2016	2.3	0.05	1.18
0.30	400	2088	2.7	0.12	1.26
0.30	350	2096	5.4	0.12	1.26
0.30	300	2122	8.2	0.12	1.26
0.30	250	2110	17.1	0.12	1.26

## Appendix B

In order to investigate the effect of process-related parameters on the penetration behavior of the cement pastes, a) the speed of the gantry was varied (1000 mm/min, 2000 mm/min, 3000 mm/min) for a constant application volume of 5.54∙10^−5^ m^3^, as well as b) the application volume (2.92∙10^−5^ m^3^, 5.54∙10 ^5^ m^3^, 8.54∙10^−5^ m^3^) for a constant gantry speed of 2000 mm/min (see Table A2). For these tests, a cement paste with a w/c ratio of 0.3 and a mini slump flow of 300 mm was used.

**Table A2:** Varied process parameters in the penetration depth tests.

Series	Variation	Volume V for 0.25 m	Gantry Speed v
-	-	in m^3^	in mm/min
(a)	1	5.54 × 10^−5^	1000
(a)	2	5.54 × 10^−5^	2000
(a)	3	5.54 × 10^−5^	3000
(b)	1	2.92 × 10^−5^	2000
(b)	2	5.54 × 10^−5^	2000
(b)	3	8.32 × 10^−5^	2000

Figure A1a shows the results of the variation of the gantry speed. It can be seen that the gantry speed seems to have no influence on the penetration depth. Only the penetration depth in dry aggregate with a d_50_ = 0.0016 m shows a gradient with increasing speed. However, the slope of the penetration depth lies within the scatter of the measurement results and can therefore be neglected.

Figure A1b exhibits the penetration depth depending on the applied volume of cement paste per length. Here, an increase of volume from 5.54∙10^−5^ m^3^ to 8.32∙10^−5^ m^3^ does not increase the penetration depth. However, halving the applied volume to 2.92∙10^−5^ m^3^ appears to have an influence on the penetration depth for all aggregate fractions, which increases with increasing medium grain diameter d_50_. However, for the smallest cement paste volume per length no continuous paste application was possible with the printer set up.

This leads to an inconsistent penetration front and a probably changed propagation behavior. Therefore, this value is not considered to be usable, as a continuous application is aimed.

Thus, the gantry speed does not represent an input parameter for the analytical calculation model. However, especially for larger applied amounts of cement paste the used volume per length should nevertheless be taken into account.

## Appendix C

In [34] the effective hydrostatic pressure was approximated by the final height h(R) of the cement paste after a mini slump flow test following [50,51] which is possible for the given test setup because the final height is mainly driven by the yield stress (see Figure A2).

## Appendix D

In this appendix, the effect of the pore system on the calculation of r_eff_ and thus on the determination of the penetration depth e is shown. For this purpose, the significance of the absolute deviation of the calculated penetration depths was calculated assuming octahedral gaps (◇) and tetrahedral gaps (∇), |∆e (◇–∇ gap)| according to model D, see Figure A3. The results for model C showed comparable results.

It can be seen that the assumption of octahedral gaps in the pore system leads to much higher penetration depths and thus to a strong overestimation. This is caused by the assumption of too large pore diameters which leads to an overestimation of the shear rates occurring in the pores and therefore an underestimation of the pressure gradient (ratio τ_0,lim_/τ_0_) as well as the flow resistance of the particle-bed.

Figure A4 shows the longitudinal cross section through of a penetration measurement. The samples exhibit that the pore system between the particles is dominated by tetrahedral gaps. Consequently, when considering the flow resistance under free flow in the β-term, the smaller and finest pores play a decisive role [47]. In contrast, the largest path between the pores determines whether or not flow begins in the pores (α-term).
